# Supplementary material for: An automated platform for high-throughput mouse behavior and physiology with voluntary head-fixation
Source: Nat Commun. 2017 Oct 30;8:1196. doi: 10.1038/s41467-017-01371-0 (PMC5662625; doi:10.1038/s41467-017-01371-0)
Supplement: Supplementary file 1 — Supplementary Information [file 41467_2017_1371_MOESM1_ESM.pdf]

Supplementary Information

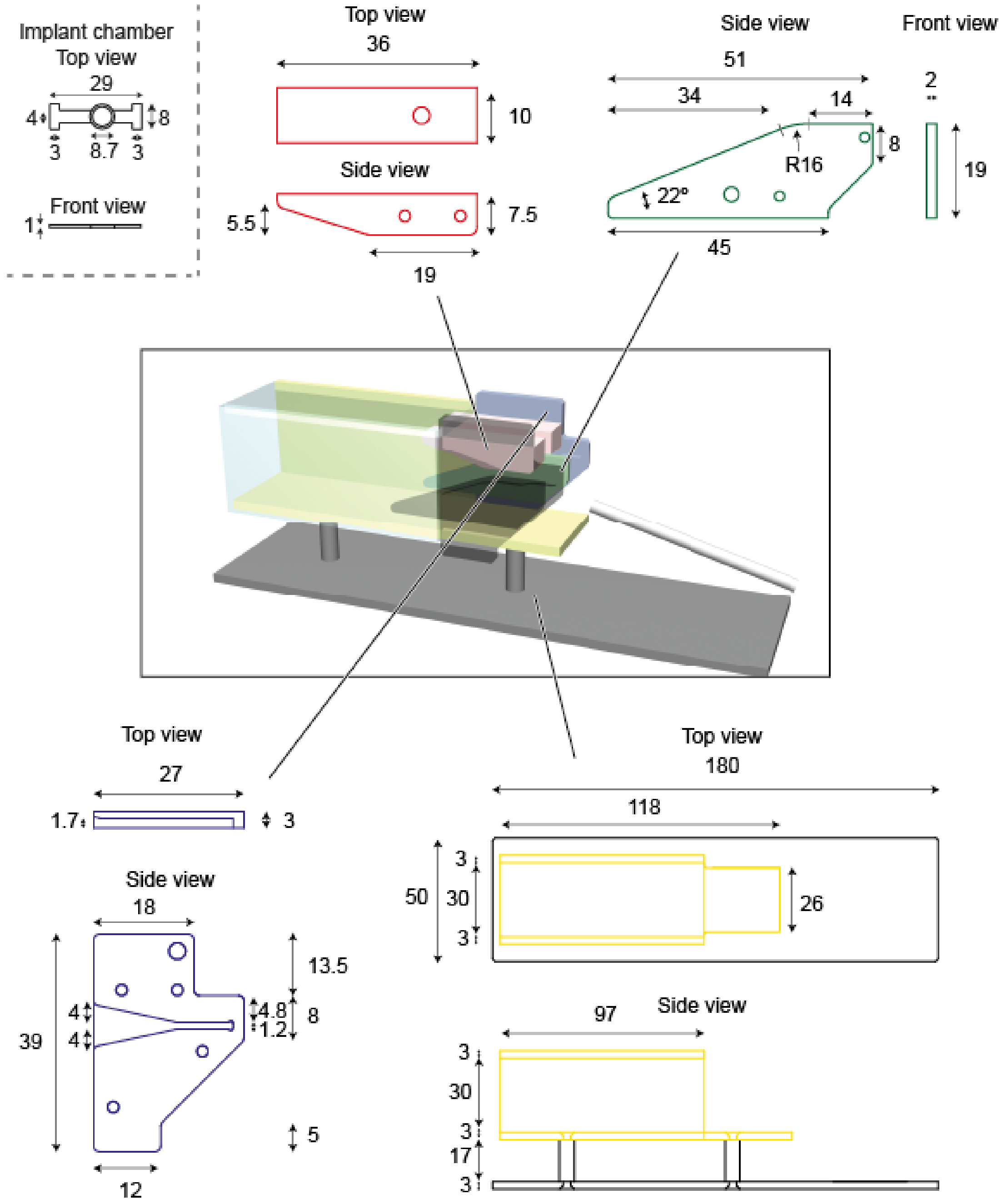

Supplementary Figure 1. Habituation tube (Fig. 1A, main text). Projections for the same part are color-coded as in the 3D rendering and in scale. Across parts projections are not in scale and the relative sizes are chosen to improve visibility. Top left inset, technical drawing of the head-post/optical-chamber implant (units in mm). Original technical drawings edited with permission from O'Hara & Co., Ltd.

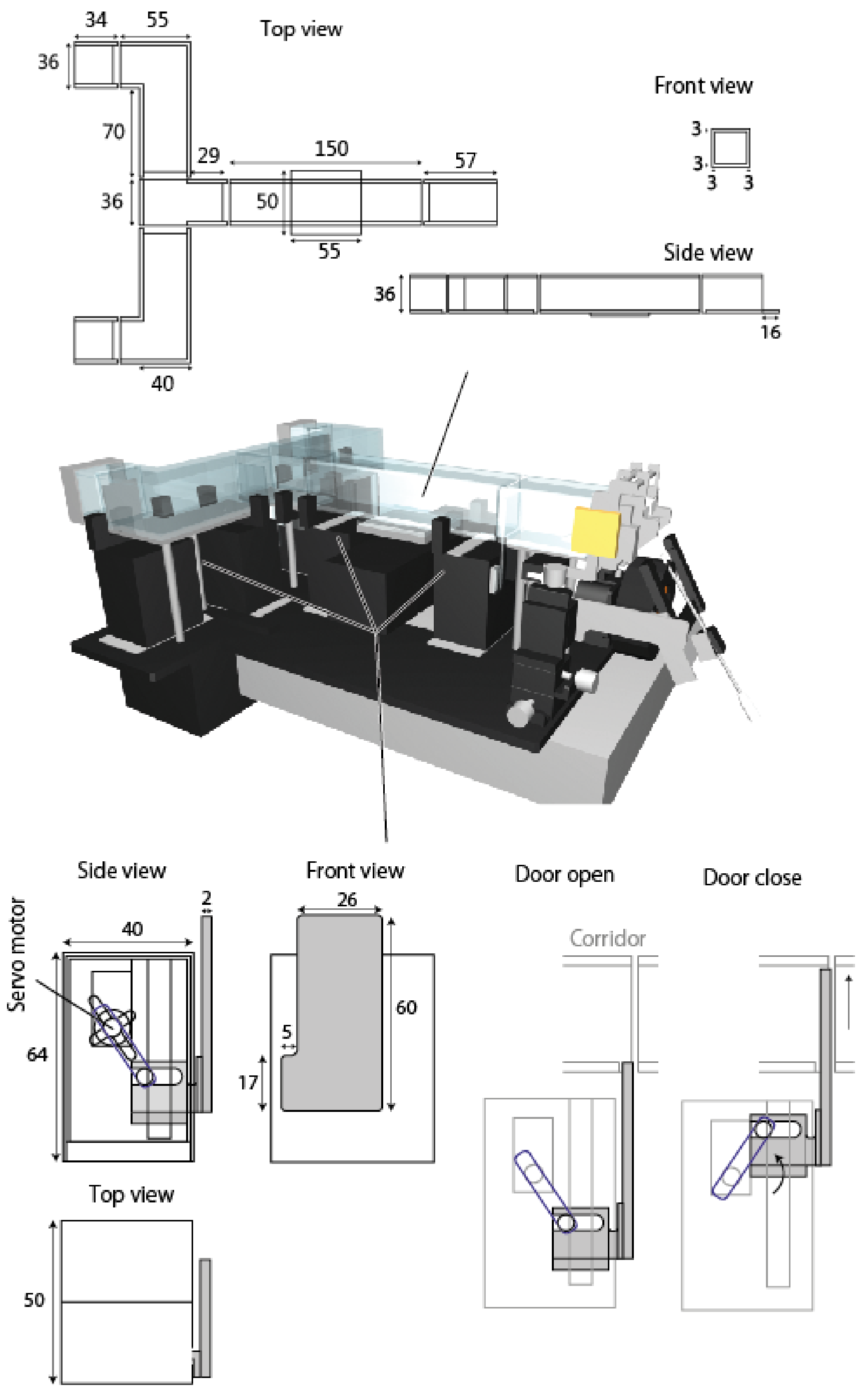

Supplementary Figure 2. Main setup, overview. Projections for the same part are in scale. Across parts projections are not in scale and the relative sizes are chosen to improve visibility. Bottom parts, servomotor actuators to control the access doors (units in mm). Original technical drawings edited with permission from O'Hara & Co., Ltd.

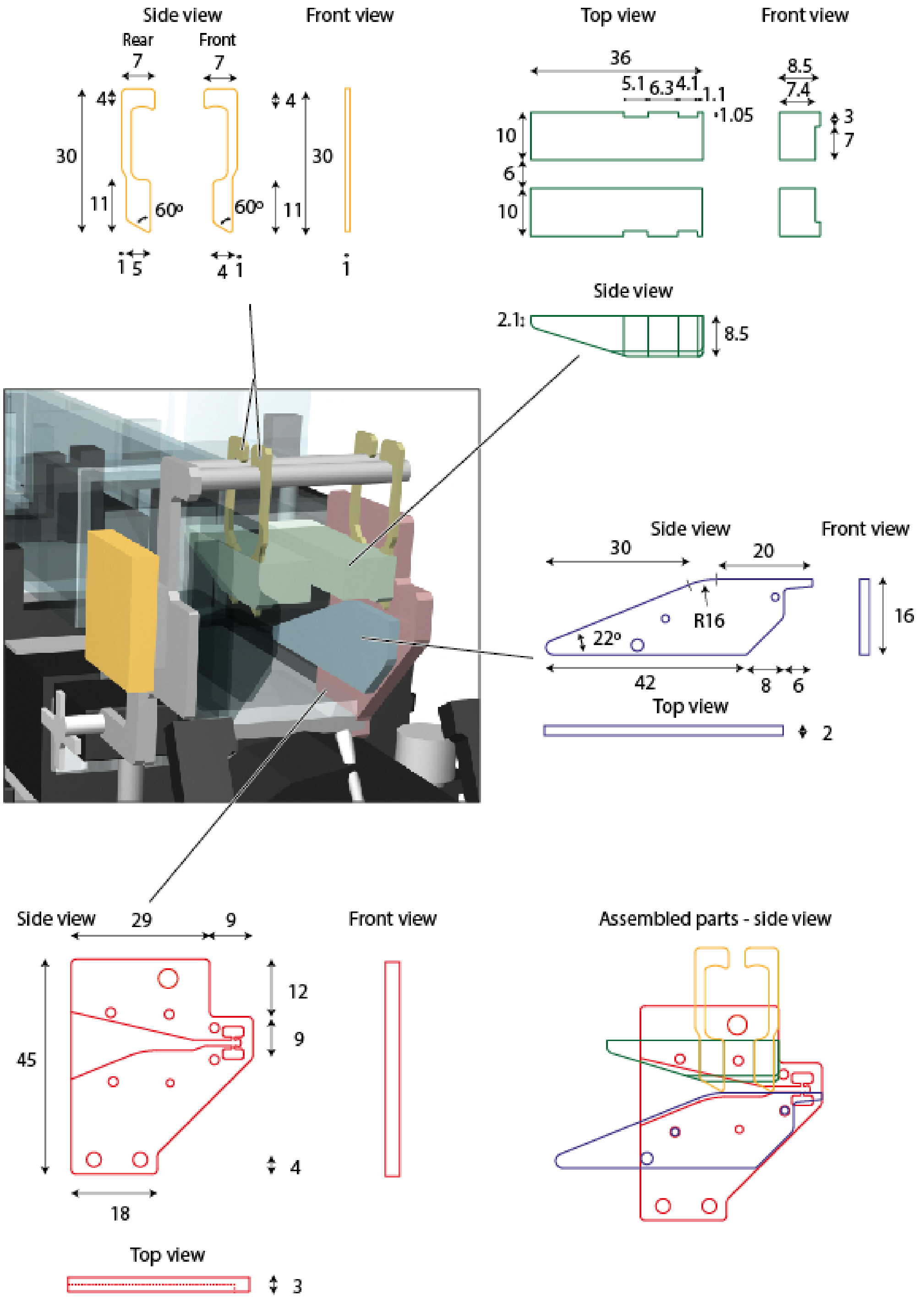

Supplementary Figure 3. Main setup, latching system. Projections for the same part are color-coded and in scale. Across parts projections are not in scale and the relative sizes are chosen to improve visibility (units in mm). Original technical drawings edited with permission from O'Hara & Co., Ltd.

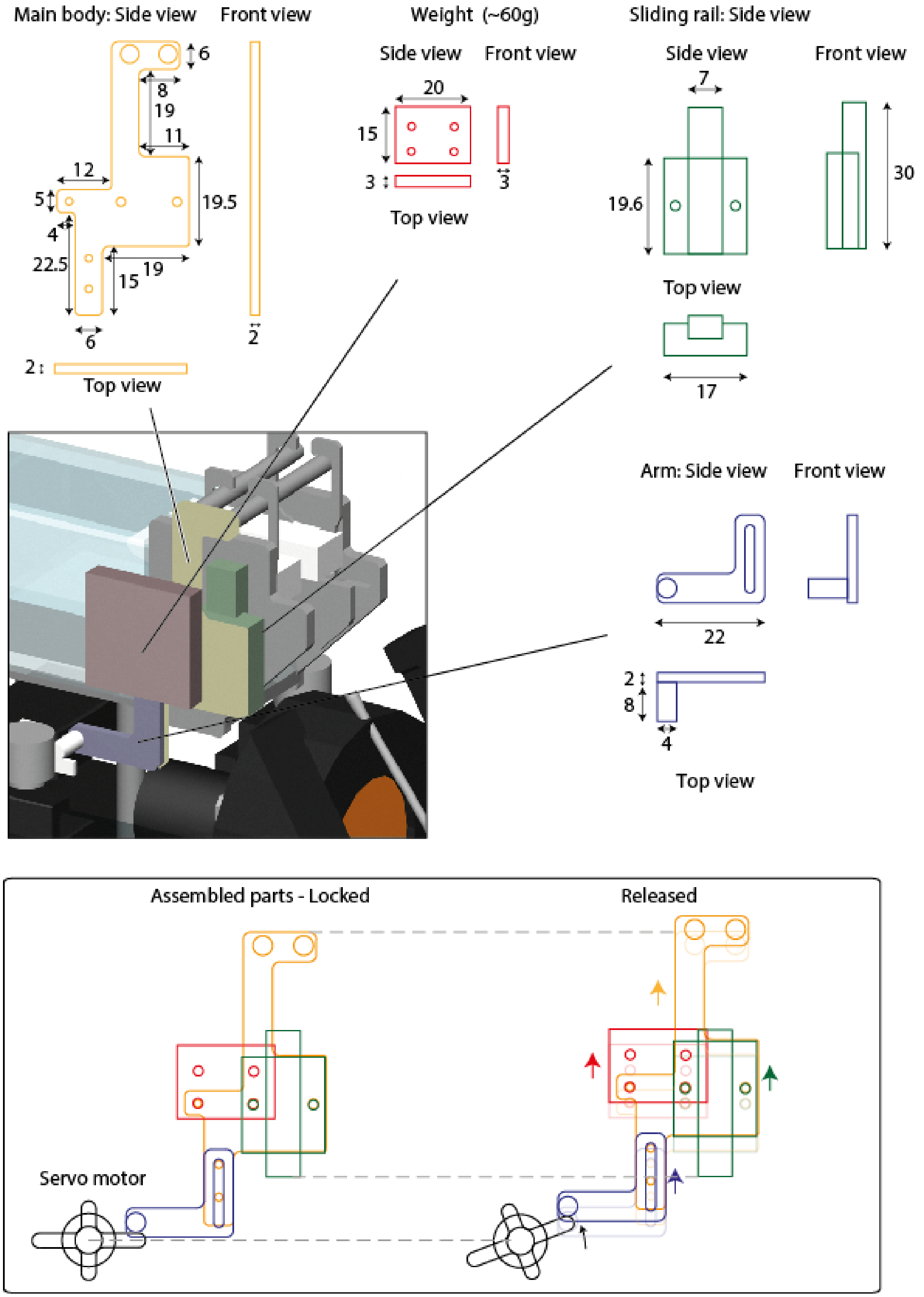

Supplementary Figure 4. Main setup, motorized unlatching mechanism. Projections for the same part are color-coded and in scale. Across parts projections are not in scale and the relative sizes are chosen to improve visibility (units in mm). Original technical drawings edited with permission from O'Hara & Co., Ltd.

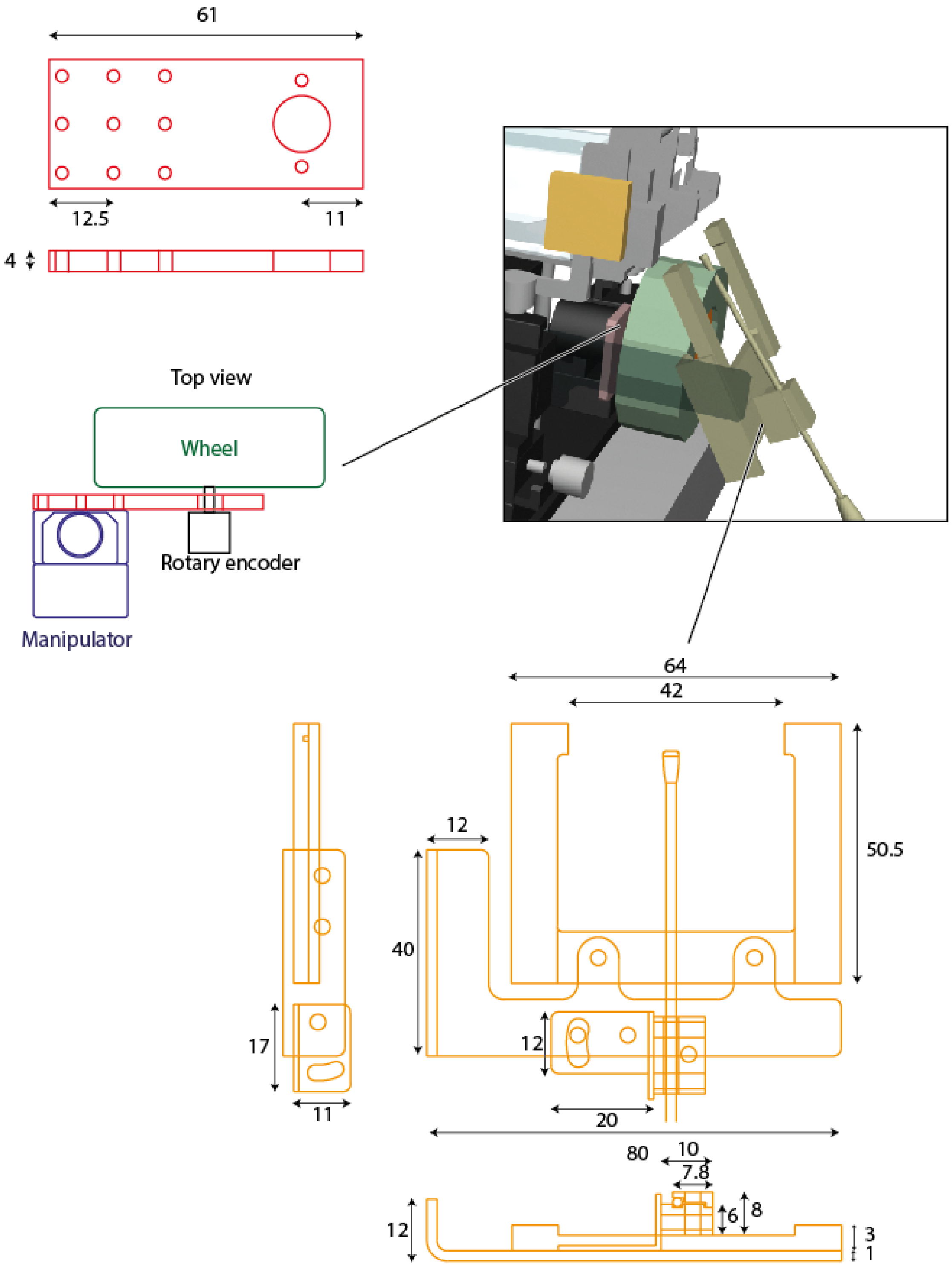

Supplementary Figure 5. Main setup, wheel and water reward system. Projections for the same part are color-coded and in scale. Across parts projections are not in scale and the relative sizes are chosen to improve visibility (units in mm). Original technical drawings edited with permission from O'Hara & Co., Ltd.

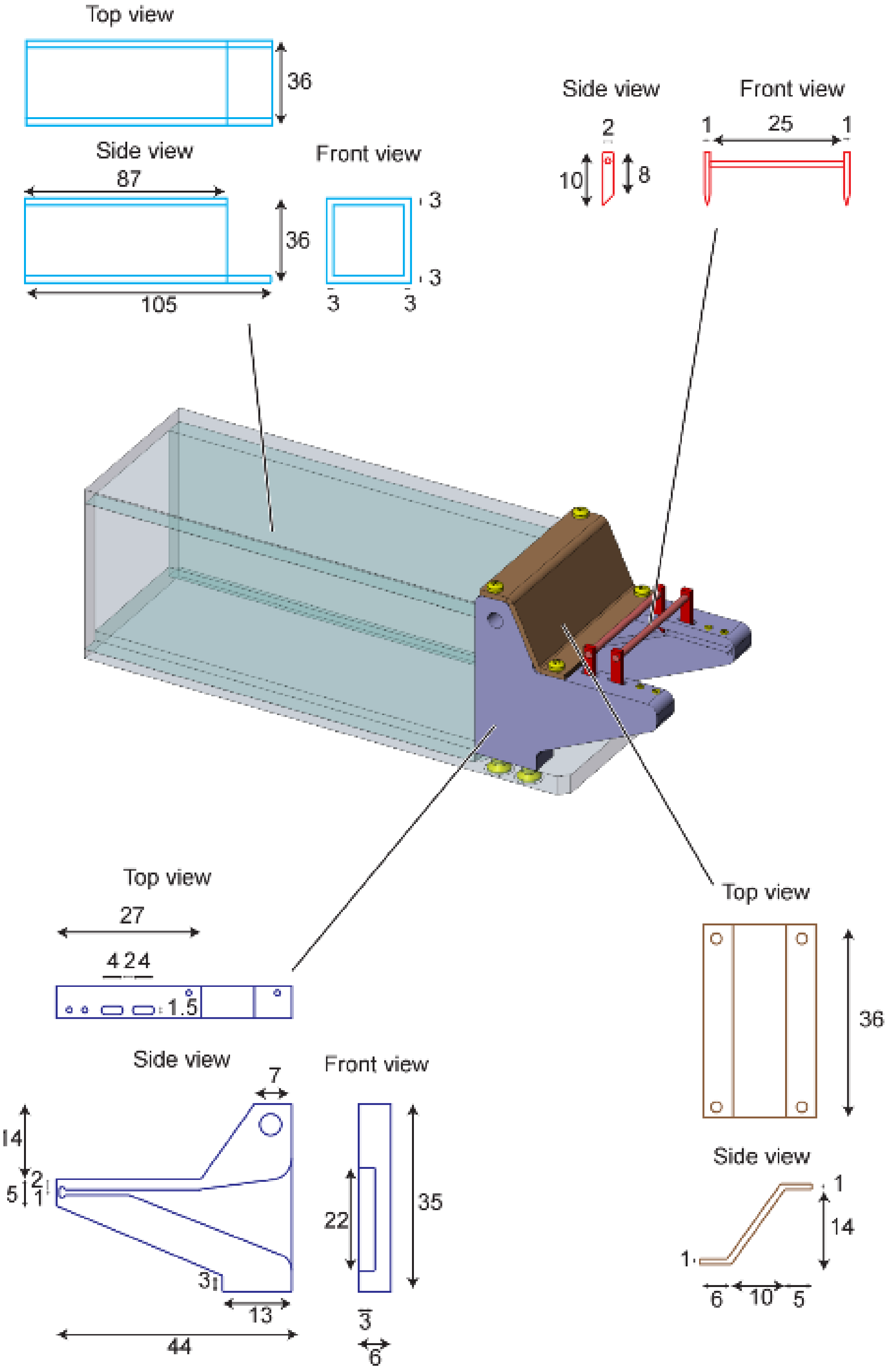

Supplementary Figure 6. Latching unit for physiology. Projections for the same part are color-coded and in scale. Across parts projections are not in scale and the relative sizes are chosen to improve visibility (units in mm). Original technical drawings edited with permission from O'Hara & Co., Ltd.

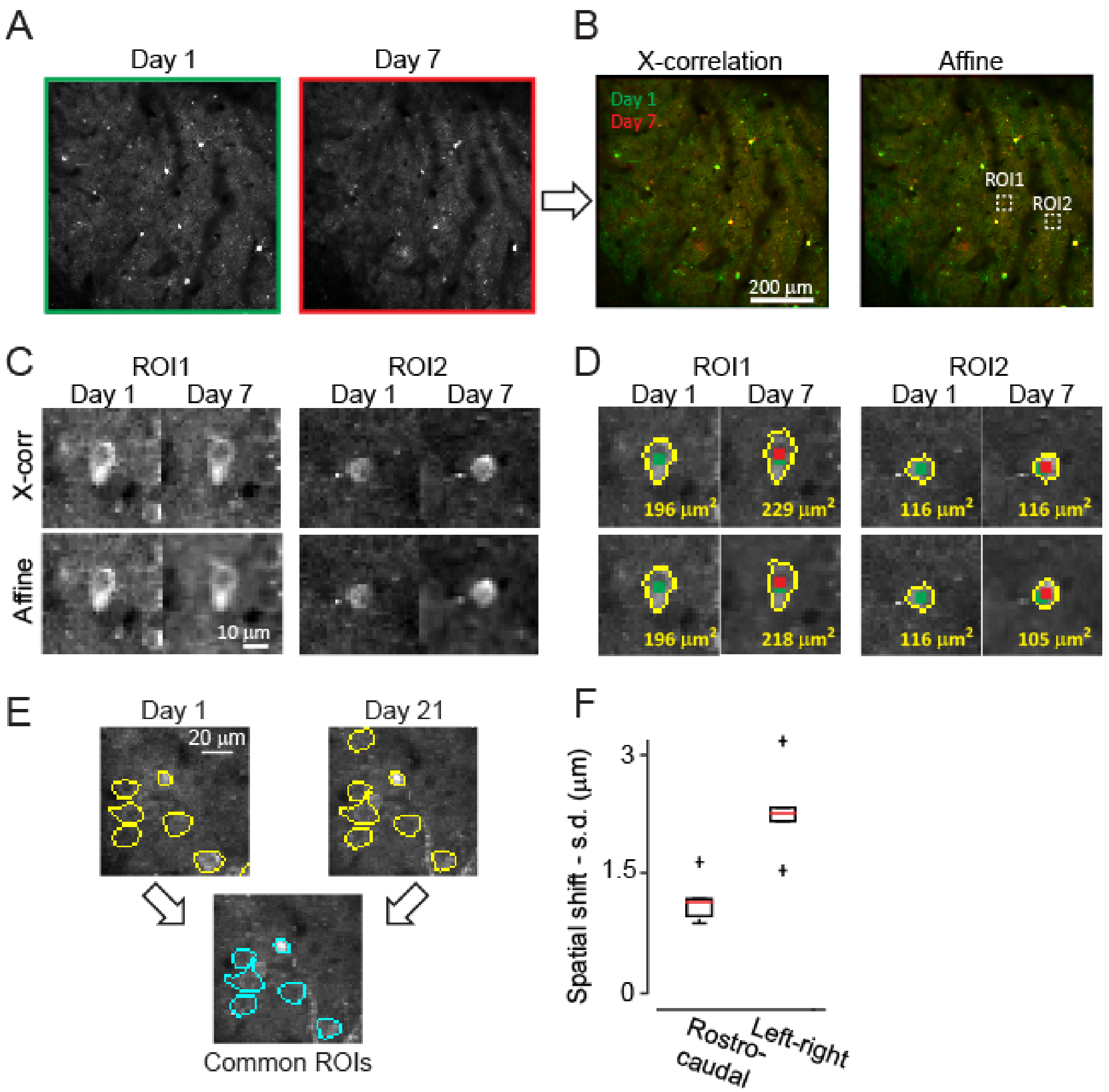

Supplementary Figure 7. Image registration across and within imaging sessions. (A) Two-photon images of GCaMP signal from two example sessions recorded one-week apart. (B) Registration of the image pair shown in A by a cross-correlation analysis (left, see Methods) or using an affine transformation method with an optimizer's metric based on Mattes mutual information, and correcting for scaling, rotation, and possible shear ('imregister' function in Matlab Image Processing Toolbox) (right). Images from day-1 and day-7 are shown in green and red color, respectively. To compare the registration precision between the two methods, we estimated the overlapping regions with strong overlapping color (yellow) pixels (> 95th percentile of each image intensity). The size of the overlapping regions were comparable for different methods: cross-correlation, 1.1x10^{4} µm^{2}, affine, 1.3 x10^{4} µm^{2}. Scale bar, 200 µm. (C) Magnified views of two rectangular ROIs indicated in B. Scale bar, 10 µm. (D) Contours of neurons were detected in each image using an edge detection algorithm ('edge' function in Matlab Image Processing Toolbox). Green and red dots indicate the center-of-masses of contoured regions in day-1 and day-7, respectively (plotted on top of each other on day-7 panels). Visual inspection of these representative examples indicates that registration errors are typically smaller than half the size of a cell's soma for both registrations methods. The number in yellow indicates the surface area of the

contoured regions. Comparable areas between two sessions support the conclusion that two images were obtained at comparable cortical depths. (E) Description of how to identify the same neurons across days (example from recordings three-weeks apart). A semi-automated segmentation algorithm (see Methods) was used independently in each imaging session. Then ROIs were matched across different sessions based on their overlap (area > 0.5 × area in the former session). Scale bar, 20 µm. (F) The within-session variability is quantified as the standard deviation of frame-by-frame translational shifts within individual sessions, according to a spatial cross-correlation analysis for image registration (Methods) (n = 6 sessions).

Supplementary Table 1.Pre-training schedule.

Genotyping	Implantation	Recovery	Habituation	Water control
Day 10-12	Day 45	(2-3 days)	(1-2 weeks)	(1 week~)

Supplementary Table 2.

CATEGORY	DESCRIPTION	COMPANY, PRODUCT CODE, (MATERIAL)	NUMBER OF ITEMS
Wheel	Wheel	LEGO 7291	1
	Rotary encoder	MICROTECH MES-12-1000PC	1
	Manipulator	Tsukumo Engineering, X15-001	1
Licking port	Licking nozzle	Fuchigami disposable zonde #140510	1
	Holder	O'Hara & Co., Ltd. (POM)	1
	IR transmitter	InfraRED LED 940nm ϕ3mm	1
	IR receiver	Photo TR ϕ3mm 940nm(peak)	1
	Micro manipulator	Tsukumo Engineering, X15-001	1
Door	Servo motor	Futaba S3114	7
	Door	O'Hara & Co., Ltd. (duralumin)	6
Corridor	Scale unit	A&D EK-6100i, customized by O'Hara & Co., Ltd.	1
	Tunnel tube	O'Hara & Co., Ltd. (PVC)	1
	IR transmitter	InfraRED LED 940nm ϕ3mm	7
	IR receiver	Photo TR ϕ3mm 940nm(peak)	7
Latching	Head fix Rail Side	O'Hara & Co., Ltd. (duralumin)	2
	Head fix Rail Top	O'Hara & Co., Ltd. (POM)	4
	IR sensor	ROHM RPI-352	1
	Servo motor	Futaba S3114	1
	Weight	O'Hara & Co., Ltd. (brass)	1
	Front pin	O'Hara & Co., Ltd. (duralumin)	2
	Rear pin	O'Hara & Co., Ltd. (duralumin)	2
Base	Base plate	O'Hara & Co., Ltd. (duralumin)	1
Latching unit for physiology	Tunnel	Custom made (acrylic)	1
	Metal wall	Custom made (aluminum)	1 pair
	Metal lid	Custom made (aluminum)	1
	Latching pin	Custom made (stainless steel)	2
Habituation tube	Tunnel	Custom made (PVC)	1
	Metal wall	Custom made (duralumin)	1 pair
	Head fix Rail Side	Custom made (duralumin)	2
	Head fix Rail Top	Custom made (POM)	2
	Base plate	Custom made (POM)	1
	Water tank	Custom made (stainless steel)	1
	Water tank lid	Custom made (stainless steel)	1
	Tube	silicon rubber, ID:4, OD:7	1
	Tube guard spring	O'Hara & Co., Ltd. (stainless steel)	1
	Nozzle	O'Hara & Co., Ltd. (stainless steel)	1

Main setup components. List of hardware components used in the habituation system, main setup, and latching unit for physiology. Several components have been designed and produced by O'Hara & Co., Ltd. under our instructions. Custom made parts have been produced in RIKEN's machine shop and only the material is indicated. Supplementary figures show the technical drawings for each component.

Supplementary Table 3.

```
CORRIDOR elements:
*Corridor doors
*Corridor sensors

INTERFACE elements:
*Rear door
*Front door
*sensor A
*sensor B
*Head Latch system

// ----------------------------------------------

Entry Procedure
        Open Corridor doors
        Open Rear door
        Close Front door
        Activate Head Latch system
        Wait for trigger(sensor A)
                If trigger, start Weight Measurement Procedure
                If timeout, start Exit Procedure
Weight Measurement
        Close rear door
        Measure weight
        Open Rear door
        Open Front door
        Start Head Latch Procedure
Head Latch
        Wait for trigger(Head Latch sensor)
                if trigger, start Session Procedure
                if timeout, start Exit Procedure
Session
        Send Message(START)
        Wait for Message(STOP)
                if Message received, start Exit Procedure
Exit
        Deactivate Head Latch system
        Wait for release(sensor A) & release(sensor B) & release(Head Latch
sensor)
                If timeout(Head Latch sensor), start Emergency Procedure
        Close Rear door
        Wait for release(Corridor sensors)
        Close Corridor doors
Sleep
        Loop
                Check Schedule
                If Active Schedule found, start Entry Procedure
                Else Check All sensors
                        If Any trigger, start Exit Procedure
        Until user signal(STOP)
```

Pseudo code for the main controller. Pseudo code for the main controller. After initialization of the main hardware components, the software relies on IR beams (sensors) to monitor the position of the animal in the setup. At each stage the software reads data from hardware components (e.g. the scale) and activates accordingly motor components (e.g. doors and the unlatching servomotor actuator). Importantly, if the head-latch sensor times out, release emergency is activated, releasing the animals and opening all doors.
