# Supplementary material for: An automated platform for high-throughput mouse behavior and physiology with voluntary head-fixation
Source: Nat Commun. 2017 Oct 30;8:1196. doi: 10.1038/s41467-017-01371-0 (PMC5662625; doi:10.1038/s41467-017-01371-0)
Supplement: Supplementary file 3 — Description of Additional Supplementary Information [file 41467_2017_1371_MOESM3_ESM.pdf]

Description of Additional Supplementary Files

File Name: Supplementary Movie 1
Description: Video demonstrating various stages for automated behavioral training: 1) the mouse is given access to the main setup directly from the home cage, 2) weight measurement is collected, 3) the mouse self-latches, 4) the training session begins and water reward is provided based on performance, 5) Finally the animal is unlatched and returns to the home cage.”
